# Single Word Reading in the Real World: Effects of Transposed-Letters

**DOI:** 10.5334/joc.160

**Published:** 2021-05-19

**Authors:** Jonathan Mirault, Jonathan Grainger

**Affiliations:** 1 Aix-Marseille University & Centre National de la Recherche Scientifique, Laboratoire de Psychologie Cognitive, Marseille, France

**Keywords:** transposed-letter effects, road sign reading, lexical decision, long-range reading

## Abstract

We investigated single word reading in a context where isolated word presentation is not unusual reading road signs in a simulated car driving situation. Participants had to indicate if the inscription on the road sign was a real word or not (lexical decision). The critical nonwords were created in two ways: by transposing two letters in a real word (e.g., the word Highway becomes Hihgway) or by substituting the same two letters with different letters (Hifpway). The baseword used to create the critical nonword stimuli were either congruent with a driving context (e.g., highway) or incongruent (e.g., garden). Nonwords created by a letter transposition were harder to reject as such compared with letter substitution nonwords a transposed-letter effect. The effect of baseword congruency was not significant and did not interact with the transposed-letter effect.

## Introduction

Much reading research has focused on single word reading, yet the vast majority of daily reading activity involves text composed of sentences. One exception to this is the reading involved in getting information from adverts, signs, etc. on billboards and other means of providing text at a distance. Road signs, for example, often use single words such as exit or stop, and driving directions are most often provided in the form of single word proper names. This context therefore provides us with the opportunity to investigate single word reading when this is the norm rather than the exception. It also allows us to investigate the extent to which laboratory studies of single word reading inform about reading in the real world (see Li et al., 2020, for a similar analysis of effects of visual crowding).

In the present study we chose to focus on one of the most robust phenomena observed in single word reading in the laboratory the transposed-letter effect (see Bruner & ODowd, 1958, for an early investigation, Grainger, 2008, for a summary of the early evidence and Grainger & Whitney, 2004, for an appraisal of its theoretical significance). The key result, to be exploited in the present work, is that in a lexical decision experiment (speeded word nonword classification), nonwords that are created from a real word by transposing two letters (e.g., *gadren* from *garden*) are particularly difficult to identify as such (e.g., Frankish & Turner, 2007). Transposed-letter effects have been observed in numerous laboratory studies using different measures and tasks (e.g., Perea & Lupker, 2003; Grainger et al., 2006; Rayner et al., 2006). One particularly important manipulation, to be applied in the present study, is the contrast between transposed-letter nonwords (e.g., *gadren*) and matched nonwords created by substituting different letters rather than transposing two letters in the same baseword (e.g., *gatsen* from *garden*) in an unprimed lexical decision task. Response times and error rates are higher for the transposed-letter nonwords compared with the double-substitution nonwords (see Perea & Lupker, 2004, and Marcet et al., 2019, for examples of studies using this manipulation).

Here we examined transposed-letter effects in a simulated driving context in order to investigate single word reading in a situation where single word presentation is not unusual. The primary aim was therefore to examine whether a transposed-letter effect can be observed in a more realistic single word reading context. We note that transposed-letter effects have been reported in the reading of logos and brand names (Perea et al., 2021), which are typically read in isolation. Hence, we had good reason to predict that we would observe similar effects in our simulated driving context. In the present study, we further manipulated whether or not the basewords used to generate critical nonword stimuli were congruent with a driving context or not. Models of visual word recognition that include top-down contextual influences on word identification (e.g., McClelland & Rumelhart, 1981; Morton, 1969), are led to predict that contextual congruence should increase the size of transposed-letter effects. That is, a congruent context should provide top-down feedback in support of a compatible baseword, hence increasing the likelihood that a transposed-letter nonword would be interpreted as the corresponding baseword. We therefore set-out to test this prediction.

## Method

### Participants

We recruited online data from 68 French native speaker volunteers (26 males, 40 females and 2 who did not choose a gender), ranged in age from 18 to 69 years old (29 between 18 and 29 years, 10 between 30 and 39 years, 11 between 40 and 49 years, 4 between 50 and 59 years and 14 between 60 and 69 years). They were informed that data would be collected anonymously prior the beginning of the experiment. Based on the criterion of Brysbaert and Stevens (2018), given the number of items per condition we recruited 68 participants to have ample power. This was confirmed in a power analysis (Green & MacLeod, 2016) indicating a power of 100% (95% IC = 5.42) for the present design.^1^ Before starting the experiment, participants accepted an online informed-consent form. Ethics approval was obtained from the Comit de Protection des Personnes SUD-EST IV (No. 17/051).

### Design & Stimuli

We constructed two sets of 75 words (from Lexique 3.83; New et al., 2004) that were estimated to be congruent or incongruent with a car driving context. A questionnaire (created with Google Forms) was proposed to a group of native French expert readers (N = 22) in order to check the generalizability of this categorization. The 150 words were presented in random order and participants were requested to note their congruency with a car-driving context on a scale from 1 to 5 (1 = highly congruent; 5 = highly incongruent). We kept congruent/incongruent words if they had an average score respectively higher or lower than 3 (average score for congruent: M = 4.34, SD = 0.52; incongruent: M = 1.54, SD = 0.51). Based on this criterion, we replaced 4 words out of the 150 that composed the questionnaire. The two sets of words were matched in length (from 5 to 8 letters) and in frequency (congruent: M = 3.90 Zipf (van Heuven et al., 2014), SD = 0.77; incongruent: M = 3.62 Zipf, SD = 0.94; t = 1.42, *p* = 0.156). From each list of 75 words, 50 were randomly selected to serve as basewords for the experimental items (nonwords). Each of these 50 words was used to create two types of nonword: either by transposing two adjacent internal letters in the baseword (e.g., garden becomes gadren) or replacing the same two letters by different letters (respecting consonant/vowel status: e.g., garden becomes gatsen). Transpositions and substitutions never involved the first or the last letter of the baseword. The remaining 25 words from each list were used as real word fillers for the purposes of the lexical decision task. We used a Latin-square design, such that each critical item was presented once only to a given participant either in the transposition condition or in the substitution condition and tested in both conditions across different participants. The final list seen by a participant was composed of 50 transposed nonwords, 50 substituted nonwords, and 50 words. Presentation order was randomized with a different order for each participant. The critical stimuli were the 50 transposed-letter and 50 substituted-letter nonwords.

### Apparatus

The experiment was created using Unity 3D software (Unity Technologies ApS) and converted into an online version using WebGL, then uploaded on the laboratorys server.

### Procedure

**Figure 1:**
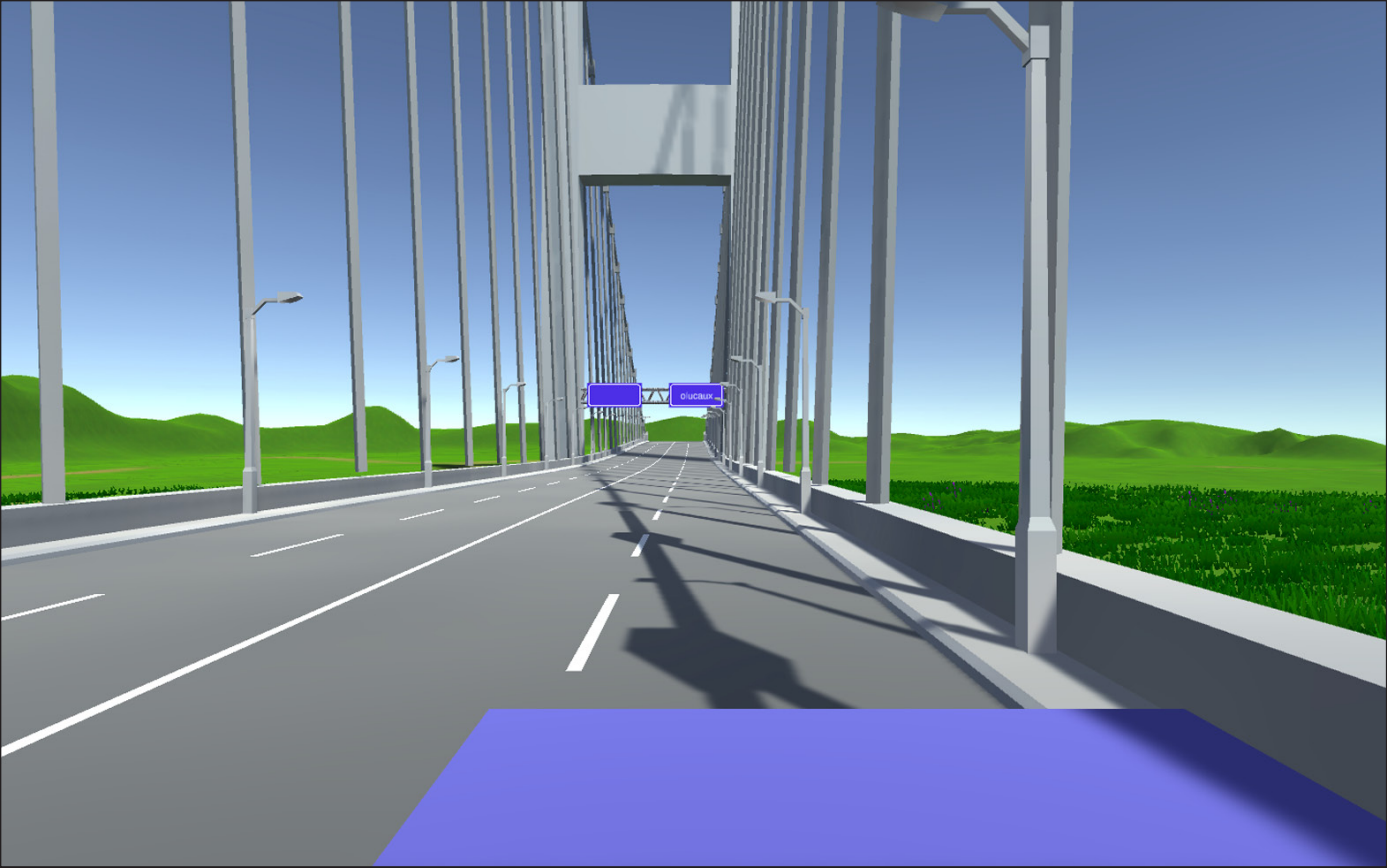
Screenshot of the beginning of one trial (just after the 3-second countdown).

At the beginning of the experiment, participants had to specify their age and their gender (anonymously) before the instructions were displayed on their personal computer in full-screen mode. When they had finished reading the instructions, they were invited to press the spacebar to start the experiment. Prior to each trial, a countdown of 3 seconds was presented (showing 3, 2, and 1 in the center of a white screen). Then, the participants point of view became a simulated car drivers point of view and moved forward at a constant velocity (28 m/s) with a standard driving scene on a highway (see ***Figure 1*** below). Experimental words and nonwords were displayed in white on a blue sign on the right side of the road (as it is typical of road signs in countries that drive on the right). Participants were instructed to press the right arrow of the keyboard if the stimulus on the road sign was a word and the left arrow if it was not a word, as rapidly and as accurately as possible.

### Dependent measures

We measured error rates and response times (RTs: time from trial onset to participants response) in making lexical decisions to the word and nonword stimuli that appeared on the highway sign.

### Analysis

We used Linear Mixed-Effects models (LMEs) to analyze our data, with items and participants as crossed random effects (including by-item and by-participant random intercepts) (Baayen et al., 2008) and with random slopes (Barr et al., 2013). Generalized (logistic) Linear Mixed-Effects model (GLME) was used to analyze errors and LMEs were used to analyze RTs. The models were fitted with the lmer (for LMEs) and glmer (for GLME) functions from the lme4 package (Bates et al., 2015) in the R statistical computing environment (R Core Team, 2018). The main factors were Transposition (transposed-letter vs. substituted-letter nonwords) and Congruency (congruent vs. incongruent baseword). We also entered Baseword Frequency (Zipf values) as a continuous covariable. We report regression coefficients (*b*), standard errors (SE) and t-values (for LMEs) or z-values (for GLME). Fixed effects were deemed reliable if |t| or |z| > 1.96 (Baayen, 2008). RTs were inverse transformed (1000/duration) prior to analysis for the purpose of normalization.

## Results

All participants performed with 75% accuracy or above. Prior to analysis, we excluded 0.26% of trials with extreme values in response times (i.e., <100 ms or >3,000 ms). The remaining dataset was composed of 10,173 observations.

### Error rate (ER)

We found a significant effect of the Transposition factor (b = 2.47; SE = 0.50; z = **4.91**) with more errors made to transposition nonwords compared to substitution nonwords. The effect of Congruency was not significant (b = 0.56; SE = 0.55; z = 1.02), and the interaction between Transposition and Congruency was not significant (b = 0.16; SE = 0.69; z = 0.24). The effect of Baseword Frequency was not significant (b = 0.25; SE = 0.19; z = 1.33). Means and 95% CIs for each experimental condition are reported in ***Table 1***.

**Table 1:** Error rates (in %) for the transposition and substitution nonwords derived from congruent and incongruent basewords.


	SUBSTITUTION	TRANSPOSITION	*EFFECT*

Congruent	1.54 (2.92)	15.64 (8.63)	*14.10*

Incongruent	1.10 (2.48)	11.34 (7.54)	*10.24*


### Response time (RT)

Prior to analysis, we excluded trials with incorrect responses (8.25%) from the data and trials with values beyond 2.5 standard deviations from the grand mean (1.27%). The remaining dataset was composed of 9,214 observations. We found a significant effect of the Transposition factor (b = 0.05; SE = 0.01; t = **3.73**), with longer RTs to transposition nonwords compared to substitution nonwords. The main effect of Congruency was not significant (b = 0.01; SE = 0.01; t = 1.24), and neither was the interaction between Transposition and Congruency (b = 0.001; SE = 0.02; t = 0.06). The effect of Baseword Frequency was significant (b = 0.01; SE = 0.006; t = **2.18**), with more frequent basewords leading to faster RTs.^2^ Means and 95% CIs for each experimental condition are reported in ***Table 2***.

**Table 2:** Response times (in ms) for the transposition and substitution nonwords derived from congruent or incongruent basewords.


	SUBSTITUTION	TRANSPOSITION	*EFFECT*

Congruent	1260 (96)	1356 (104)	*96*

Incongruent	1255 (100)	1323 (100)	*68*


### Post-hoc analysis of word length and word frequency

Given that our word targets varied in length (in number of letters) and frequency, we performed post-hoc analyses entering length as a continuous variable in LME and GLME analyses performed on the error rates and RTs to word targets (N = 50). Word length significantly affected RTs (b = 0.01; SE = 0.00; t = **3.74**), with longer RTs to longer words. The effect of length on error rates was not significant (b = 0.10; SE = 0.11; z = 0.93). There was a significant effect of Frequency in error rates (b = 0.88; SE = 0.18; z = **4.87**) and RTs (b = 0.04; SE = 0.00; t = **4.88**). Word frequency facilitated responses to word targets in our simulated car driving context.

We also examined the effects of length on performance to the critical nonword targets, and found a significant impact of length on RTs, with longer RTs for longer nonwords (b = 0.01; SE = 0.00; t = **3.15**). The effect on error rates went in the same direction but failed to reach significance (b = 0.22; SE = 0.15; z = 1.50).

### Post-hoc analyses with a median split of baseword frequency

In order to provide a further investigation of the impact of baseword frequency on transposed-letter effects, we performed post-hoc analyses where we divided the nonword targets into two baseword frequency groups (high vs. low) using a median split. In the error rates there was a significant effect of the Transposition factor (b = 2.78; SE = 0.45; z = **6.10**), but no effect of Congruency (b = 0.60; SE = 0.52; z = 1.14) and no effect of Baseword Frequency (b = 0.24; SE = 0.50; z = 0.49). None of the interactions were significant (all z < 0.85). In the RT data there was again a significant effect of Transposition (b = 0.06; SE = 0.32; z = **2.07**), and no effect of Congruency (b = 0.02; SE = 0.02; z = 0.83) or Baseword Frequency (b = 0.02; SE = 0.02; z = 0.89). Once more the interactions were not significant (all z < 0.47).^3^

## Discussion

In the present study we aimed to further investigate a well-studied phenomenon found in laboratory studies of single word reading the transposed-letter effect in a context where single word presentation is more the norm than the exception. We presented a mix of real words and nonwords in a simulated driving context, with the stimuli presented on a highway sign and participants viewing these as if they were driving on the highway. Participants had to make lexical decision to these stimuli, and we recorded error rates and RTs. The critical stimuli were two types of nonword derived from the same baseword: transposition nonwords formed by transposing two letters in the baseword, and substitution nonwords formed by replacing the same two letters with different letters. Moreover, the basewords used to form these nonwords could either be congruent with a driving context or not. We found highly robust transposition effects in all our measures, but no significant effects of baseword congruency and no significant interaction between congruency and transposition. Significant effects of word frequency and word length were also found, as well as effects of length on responses to the critical nonword targets, although not systematically on both errors and RTs. Finally, there was some evidence for an effect of baseword frequency when this was entered as a continuous variable along with the Transposition and Congruency factors, with higher baseword frequencies leading to faster RTs to nonwords (see Perea et al., 2005, for a detailed analysis of effects of baseword frequency on responses to nonwords, with evidence that a higher baseword frequency can facilitate performance in a go/no-go lexical decision task with single-letter substitution nonwords).^4^

The main goal of the present study was to replicate a well-established laboratory finding concerning visual word recognition transposed-letter effects (e.g., Perea & Lupker, 2003; 2004; see Grainger, 2008, for a review) in conditions where single word presentation is not unusual. We were entirely successful in replicating this laboratory finding in our simulated car driving context. We also replicated the standard effects of word frequency and baseword frequency (for nonwords) on lexical decision responses. The length effect on nonword responses is also a classic finding, whereas the length effect we observed on responses to word stimuli from 5 to 8 letters in length is typically not found in standard lexical decision experiments (e.g., the analyses of large lexical decision databases: English Lexicon Project (ELP, Balota et al., 2007), Dutch Lexicon Project (DTP, Keuleers et al., 2010), British Lexicon Project (Keuleers et al., 2012), French Lexicon Project (FLP, Ferrand et al., 2010). We speculate that the atypical length effect seen with word stimuli in the present study might be due to the greater viewing distance at the beginning of trials and the corresponding drop in acuity (see ***Figure 1***). This reduced acuity could necessitate more focused attention on parts of words, resulting in sequential sampling of information rather than parallel letter identification.

Finally, we failed to find an effect of baseword congruency on responses to nonwords, and this factor did not significantly interact with transposed-letter effects, although there was a numerical trend in the expected direction. This most likely reflects the difficulty in finding a sufficient number of words that are highly constrained by a driving context, and especially with respect to what one might expect to see written on a highway sign. Given that there is some evidence that transposed-letter effects in sentence reading are modulated by prior context (Acha & Perea, 2008; Johnson, 2009) it would be interesting in future research to investigate the impact of driving context on more highly constrained words, such as place names, which frequently appear in this context. Effects of context on transposed-letter effects are predicted by interactive models of visual word recognition (e.g., McClelland & Rumelhart, 1981; Morton, 1969) according to which context should provide top-down support for the basewords that are associated with the critical nonwords, therefore making it harder to detect letter transpositions.

The present results demonstrate that laboratory studies of single word reading, that are necessarily relatively artificial simplifications of reading in the real world, do capture basic phenomenon that occur in more naturalistic reading situations. In terms of potential applications of this work, understanding the factors that determine the ease with which drivers can read road signs is important because even a few milliseconds of distraction can determine whether or not an accident occurs. Designing road signs while taking into consideration the cognitive factors known to affect reading fluency is clearly one interesting avenue for future applied research.

## Data Accessibility Statement

Hypothesis and experimental plan were pre-registered 1st of June 2020 and are available at: *https://osf.io/7xezf*. Data and materials are available at: *https://osf.io/z3spg/*.

## Footnotes

We used the SIMR package in R software for this power analysis. The power analysis ran over 100 simulations and expressed the power of the RT lmer model for the Transposition factor (independent measure).

We note, nevertheless, that when baseword frequency was analyzed separately (i.e., without the Transposition and Congruency factors) the effect was only marginally significant (t = 1.72).

An analysis using the lower and upper quartiles of baseword frequency revealed the same pattern of results.

As noted by Perea et al. (2005), such facilitatory effects of baseword frequency can be interpreted as reflecting the operation of a verification mechanism to make nonword decisions (see Grainger, 2018, for further discussion).

## Significant statement

The present research is the first to address one crucial aspect of driving the ability to read road signs efficiently with minimal time allocated to that task. Since every millisecond counts when driving, this is an important issue since reading times can vary well beyond the tens to hundreds of milliseconds. Here we simply aimed to demonstrate how carefully controlled laboratory experimentation can investigate this. We furthermore aimed to show that one particularly robust phenomenon seen in laboratory studies of single word reading can be replicated in a simulated driving context.
